# Gardening and subjective cognitive decline: a cross-sectional study and mediation analyses of 136,748 adults aged 45+ years

**DOI:** 10.1186/s12937-024-00959-9

**Published:** 2024-06-04

**Authors:** Kaiyue Wang, Yaqi Li, Xiao Chen, Susan Veldheer, Chen Wang, Han Wang, Liang Sun, Xiang Gao

**Affiliations:** 1 Department of Nutrition and Food Hygiene, School of Public Health, Institute of Nutrition, Fudan University, Shanghai, 200032 China; 2 Departments of Family and Community Medicine and Public Health Sciences, Penn State College of Medicine, Hershey, PA USA; 3 Shanghai Institute of Sports Science, Shanghai, 200030 China; 4 Department of Neurology, Peking Union Medical College Hospital, Chinese Academy of Medical Sciences, Beijing, 100730 China

**Keywords:** Gardening, Physical activity, Cognitive decline, Functional limitations

## Abstract

**Background:**

Given the benefits of gardening for physical and psychological health, we explored whether gardening was associated with lower risks of subjective cognitive decline (SCD), a precursor of dementia, and SCD-related functional limitations.

**Methods:**

Included in this cross-sectional study were 136,748 participants aged 45 + years old from the Behavioral Risk Factor Surveillance System 2019 survey, who were then categorized into three groups according to self-reported exercise status: non-exercisers, gardeners, and other exercisers. SCD was assessed via a questionnaire, and SCD-related functional limitations were referred to as having difficulties in engaging in household or social activities due to SCD. The odds ratio (OR) and 95% confidence interval (CI) were calculated to assess the associations of gardening with SCD and SCD-related functional limitations, adjusted for age, sex, socioeconomic status, lifestyle factors, and health status. Mediation analyses were conducted to examine whether the observed association between gardening and SCD was mediated by energy expenditure (MET-hours/week), depression status, and consumption of fruits and vegetables.

**Results:**

Overall, 11.1% and 5.4% of participants self-reported experiencing SCD and SCD-related functional limitations, respectively. The adjusted OR for gardeners vs. non-exercisers, was 0.72 (95% CI 0.62–0.83) for SCD and 0.57 (95% CI 0.44–0.73) for SCD-related functional limitations. The observed association between gardening and SCD was explained by higher energy expenditure (39.0%), lower likelihood of having depression (21.5%), and higher consumption of fruits and vegetables (3.4%) (*P*<0.05 for all). Similar patterns were observed for SCD-related functional limitations.

**Conclusion:**

In this nationally representative sample, gardening was associated with better cognitive status, which may be mainly attributed to better depression status and energy expenditure.

**Supplementary Information:**

The online version contains supplementary material available at 10.1186/s12937-024-00959-9.

## Introduction

Subjective cognitive decline (SCD) is a syndrome characterized by subjective memory decline without a diagnosis of mild cognitive impairment (MCI), which required objective evidence of cognitive impairment from clinical and neuropsychological examinations [1]. However, increasing evidence suggested that SCD was a preclinical stage of dementia progression, and individuals with SCD had a heightened probability of developing MCI and dementia compared to those without SCD [2–6]. In parallel, limitations in functional capacities are of unique importance in reflecting the progression from cognitive decline to dementia [7–9]. Early interventions/therapies thus served to resist the pathogenic progression of cognitive decline. Non-pharmaceutical interventions, such as aerobic physical activity (PA) [10] and horticultural therapy [11], gained increasing attention and were carried out for slowing this decline, although the effectiveness remained controversial [12–14].

According to Physical Activity Guideline for Americans (2nd edition) [15], gardening is a muscle-strengthening and multicomponent PA with one of the lowest injury risks, which is appropriate and recommended for older adults. Of particular note were the benefits of gardening for mental health and well-being, including potential reductions in anxiety and depression symptoms [16, 17] and improved sleep quality [18, 19]. We previously reported that people who engaged in gardening had a lower frequency of mental distress and lower odds of diabetes [20], a known risk factor of dementia [21]. Previous cohort studies reported that gardening activity was associated with lower risk of dementia [22–25], however, those studies were limited by relatively small sample size (< 5000 for all) and they did not control several important covariates, such as demographic characteristics (e.g. age, sex) [24], education levels [22], and lifestyle factors (e.g. smoking, drinking, and obesity) [22–25]. Other small-scale clinical studies also showed that gardening could improve symptoms of cognitive decline among individuals with dementia [26, 27]. However, the aforementioned studies focused more on dementia and cognitive impairment rather than on SCD. To date, no large-scale population-based study has examined whether gardening is associated with SCD, a crucial time window for dementia prevention. Further, it remained unclear to what extent the association between gardening and cognitive function could be explained by some of the crucial mediators, such as PA levels, mental health, and dietary intake. This is the key to understanding the underlying mechanisms of SCD so that intervention strategies can be optimized accordingly.

Therefore, we examined the associations between gardening and risk of SCD and SCD-related functional limitations in a nationally representative sample of ~ 140,000 participants in the United States (US). To explore the potential pathways underlying the gardening-cognition relationship, we further examined whether energy expenditure, depressive status, and consumption of fruits and vegetables mediated the association between gardening and cognitive function.

## Methods

### Study design and population

The Behavioral Risk Factor Surveillance System (BRFSS) is a nationally representative survey, conducted annually by US state and territory health departments. State-level data were collected using the standardized core questionnaire (plus optional modules and state-added questions), as described elsewhere [28–30]. We used the publicly available de-identified BRFSS data from the 32 states that carried out cognitive decline module survey in 2019 (*n* = 225,150, Alabama, Connecticut, District of Columbia, Florida, Georgia, Indiana, Iowa, Louisiana, Minnesota, Mississippi, Missouri, Nevada, New Mexico, North Dakota, Oregon, Pennsylvania, Rhode Island, South Carolina, South Dakota, Tennessee, Texas, Virginia, West Virginia, Wisconsin, Michigan, Nebraska, Ohio, Utah, Kansas, New York, Oklahoma, and Maryland). The cognitive decline module was performed among participants aged ≥ 45 years old. Exclusion of participants with incomplete information on PA types (*n* = 15,670), experiencing SCD (*n* = 71,775), SCD-related functional limitations (*n* = 325), and depression (*n* = 632) resulted in 136,748 participants eligible for analyzing with SCD and SCD-related functional limitations as outcomes.

### Assessments of gardening and physical activity levels

Engaging in PA was defined by the question “During the past month, other than your regular job, did you participate in any physical activity or exercise such as running, calisthenics, golf, gardening, or walking for exercise?”. If individuals responded ‘Yes’, they were asked about the types of PA in which they engaged during the previous month. From a list of 75 activities, participants chose the first and second most time-consumed activities and the corresponding frequency and duration of each activity was recorded. Consistent with our previous research, gardeners were identified as those who selected gardening as the first or second activity [20]. Non-exercisers reported no PA and other exercisers engaged in first and second activities other than gardening.

The PA compendium from the BRFSS calculated a series of variables for evaluation of PA levels of the participants [31]. Initially, metabolic equivalent (MET) values were assigned to the 75 activities in the list and the estimation of functional capacity (FC) was obtained from 60% maximum oxygen consumption. The relative intensity of each activity of individuals was classified as ‘vigorous’ (MET values ≥ FC/100), ‘moderate’ (MET values ≥ 3.0), and ‘light or not aerobic’ (MET values ≥ 0). Next, the calculation of exercise time (min/week) was calculated from frequency and duration of each activity. Based on weekly duration of moderate-intensity or vigorous-intensity PA, PA levels were further subdivided into four classes: ‘Inactive’ (no PA), ‘Insufficiently active’ (1-149 minutes of moderate-intensity activities), ‘Active’ (150–299 minutes of moderate-intensity activities), ‘Highly Active’ (>300 minutes of moderate-intensity activities or half of vigorous-intensity activities), in accordance with the Physical Activity Guideline for Americans (2nd edition) [15].

### Assessments of SCD and SCD-related functional limitations

Participants were considered as SCD cases if they answered ‘Yes’ to the question, “During the past 12 months, have you experienced confusion or memory loss that is happening more often or is getting worse?”. Individuals with SCD were further asked the two questions regarding functional limitations: “During the past 12 months, as a result of confusion or memory loss, how often have you given up day-to-day household activities or chores you used to do, such as cooking, cleaning, taking medications, driving, or paying bills?”, “During the past 12 months, how often has confusion or memory loss interfered with your ability to work, volunteer, or engage in social activities outside the home?”. Participants who answered ‘Always’ or ‘Usually’ or ‘Sometimes’, rather than ‘Rarely’ and ‘Never’, for either of the questions, were classified as having SCD-related functional limitations [32–34].

### Assessment of covariates

Data on demographics, lifestyle factors, and chronic health conditions were collected from the core section of the BRFSS questionnaire. Demographic information included age (<60, 60-69.9, 70-79.9, ≥ 80 years old), sex (men, women), education levels (did not graduate from high school, graduated from high school, attended college or technical school, graduated from college or technical school), marital status (married or living as married, other), race (non-Hispanic White, non-Hispanic Black, Hispanic, other), annual household income (<$15,000, $15,000–49,999, ≥$50,000), and body mass index (BMI, weight in kg/height in meters [2]. Current smokers were participants who self-reported having smoked at least 100 cigarettes in their lifetime and currently smoked. Current drinkers were those who had consumed at least one alcoholic beverage in the past 30 days. Regarding chronic health conditions, the presence of diabetes and hypertension were included in the analyses as covariates. Missing data from categorical variables were classified as a separate group, while missing data for continuous variables were re-encoded with the median.

### Potential mediators

The mediation analyses were conducted to examine to what extent the gardening-cognition relationship could be explained by energy expenditure, depression status, and fruits and vegetables consumption. Energy expenditure was calculated from the MET value of each activity and the corresponding weekly duration (MET-hours/week). The assessment of psychological and mental well-being status was to answer a question about depressive disorder. Participants who reported ‘Yes’ to the question “Ever told you had a depressive disorder (including depression, major depression, dysthymia, or minor depression)?” was classified as experiencing depressive disorder. Further, the BFRSS investigated the frequency of participants’ daily intake of fruits, dark green vegetables, potatoes, and other vegetables. The consumption of fruits and vegetables was calculated as the sum of the four items above.

### Statistical analysis

All data were analyzed using SAS software (version 9.4, SAS Institute Inc, USA). *P* < 0.05 was considered as statistically significant. General characteristics of study population was expressed as means ± standard deviation (SD) for continuous variables and unweighted number (weighted percentage) for categorical variables. Based on the analysis guide of complex sampling data from BRFSS [35], logistic regression models were developed to examine the association of SCD with gardening and non-gardening activities, respectively, included the procedures of stratification, clustering, and sample weighting, and adjusted for aforementioned covariates. Thus, the weighted odds ratios (ORs) and 95% confidence intervals (CI) were estimated. Similar analyses were conducted for SCD-related functional limitations. To test the robustness of the effect size estimation, we also calculated prevalence ratios (PRs) and corresponding 95%CI for gardening-cognition association using the generalized linear models that adjusted for the aforementioned covariates.

Mediation analyses were performed to estimate direct effect (without mediators), indirect effect (with mediators), and mediation proportion using a SAS macro %MEDIATE [36], after adjustment for aforementioned covariates. We further tested the multiplicative interactions between individual variables (age, sex, education levels, race, BMI) and gardening using likelihood ratio test.

Additionally, gardeners may be engaged in other PA, and the resulted physical exertion may affect the association between gardening and cognitive decline. Therefore, we conducted a sensitivity analysis after excluding the gardeners whose PA levels of other activities reached the ‘Active’ (≥ 150 min of moderate-intensity activities or half of vigorous-intensity activities) (*n* = 6,689). To examine the robustness of the associations in various geographic locations, we also performed a sensitivity analysis after stratifying the populations by the 32 states.

## Results

A total of 136,748 participants were included in the cross-sectional study and categorized as non-exercisers (31.2%), gardeners (8.8%), and other exercisers (60.0%), and characteristics of study participants were summarized in Table 1. Compared with non-exercisers and other exercisers, gardeners had higher proportions of the older adults ≥ 60 years old (65.0%), women (62.1%), and non-Hispanic Whites (80.3%). The prevalence of diabetes (14.2%) was lower in the gardeners than in non-exercisers and other exercisers. Also, gardeners and other exercisers ate fruits and vegetables more than 3 times a day on average.

**Table 1:** General characteristics of study population according to physical activity types, Behavioral Risk Factor Surveillance System 2019

	Non-exercisers	Gardeners	Other exercisers
**Participants**	41,846 (31.2%)	12,705 (8.8%)	82,197 (60.0%)
**Age, years, %**			
< 60	12,470 (39.7%)	3344 (33.0%)	27,815 (43.7%)
60-69.9	11,859 (27.5%)	4325 (34.0%)	25,320 (28.6%)
70-79.9	10,293 (19.5%)	3517 (22.9%)	19,161 (18.2%)
≥ 80	6683 (11.7)	1342 (8.1%)	8544 (7.2%)
Missing	541 (1.6%)	177 (2.0%)	1357 (2.3%)
**Sex, women, %**	25,038 (56.3%)	8452 (62.1%)	44,374 (50.9%)
**Education levels, %**			
Not graduate from high school	5037 (20.1%)	564 (8.1%)	4210 (9.2%)
Graduated from high school	15,460 (36.4%)	3183 (27.4%)	19,117 (24.8%)
Attended college or technical school	11,679 (27.4%)	3746 (33.0%)	22,179 (30.3%)
Graduated from college or technical school	9495 (15.6%)	5195 (31.2%)	36,479 (35.3%)
Missing	175 (0.5%)	17 (0.3%)	212 (0.4%)
**Ethnicity, %**			
Non-Hispanic White	32,604 (68.3%)	11,066 (80.3%)	67,375 (73.0%)
Non-Hispanic Black	4419 (14.0%)	581 (6.8%)	6665 (11.9%)
Hispanic	2183 (11.2%)	458 (7.3%)	3203 (8.0%)
Other	1846 (4.7%)	405 (3.8%)	3354 (5.0%)
Missing	794 (1.8%)	195 (1.8%)	1600 (2.1%)
**Married or living as married, %**	19,998 (53.4%)	7915 (67.5%)	48,821 (64.8%)
**Annual household income, %**			
<$15,000	4887 (12.3%)	631 (5.5%)	4876 (5.7%)
$15,000–49,999	17,466 (40.7%)	4279 (31.5%)	24,830 (28.7%)
≥$50,000	11,398 (27.5%)	5689 (45.2%)	39,265 (49.9%)
Missing	8095 (19.5%)	2106 (17.8%)	13,226 (15.7%)
**Body mass index, kg/m**^**2**^, **SD**	29.9 (0.30)	28.1 (0.13)	28.4 (0.04)
**Current smoker, %**	7722 (20.7%)	1690 (14.7%)	8663 (11.7%)
**Current drinker, %**	14,521 (35.9%)	6615 (52.9%)	42,231 (52.2%)
**Diabetes, %**	10,959 (25.4%)	1933 (14.2%)	13,745 (16.7%)
**Hypertension, %**	25,040 (57.0%)	6394 (48.2%)	39,714 (46.0%)
**Consumptions of fruits and vegetables, times/day, SD**	2.4 (0.03)	3.3 (0.08)	3.1 (0.03)

Data are n (%) or mean (SD)

Among all participants, 14,909 (11.1%) individuals had experienced SCD, and 6,611 (5.4%) individuals had developed functional limitations, but the prevalence of SCD and SCD-related functional limitations for gardeners and other exercisers were significantly lower than for non-exercisers (Table 2). The ORs for gardeners vs. non-exercisers were 0.72 (95% CI 0.62–0.83) for SCD and 0.57 (95% CI 0.44–0.73) for SCD-related functional limitations, adjusted for SCD risk factors. Similar patterns were observed when comparing other exercisers to non-exercisers, where the OR was 0.70 (95%CI 0.65–0.75) for SCD and 0.58 (95%CI 0.52–0.64) for SCD-related functional limitations, respectively. Similar effect sizes were observed when we used PRs to measure the association of gardening with SCD and SCD-related functional limitations (Supplemental Table 1).

**Table 2:** Odds ratios and 95% confidence interval of subjective cognitive decline (SCD) and SCD-related functional limitations according to physical activity types, Behavioral Risk Factor Surveillance System 2019

	Non-exercisers	Gardeners	Other exercisers
**Individuals with SCD / total, %** *	6357/41,846 (15.9)	1124/12,705 (9.2)	7428/82,197 (8.9)
Model 1 ^a^	1 (Ref)	0.61 (0.53, 0.70)	0.59 (0.55, 0.64)
Model 2 ^b^	1 (Ref)	0.72 (0.62, 0.83)	0.70 (0.65, 0.75)
**Individuals with SCD-related functional limitations / total, %** *	3512/41,846 (9.3)	350/12,705 (3.4)	2749/82,197 (3.6)
Model 1	1 (Ref)	0.45 (0.35, 0.57)	0.46 (0.41, 0.51)
Model 2	1 (Ref)	0.57 (0.44, 0.73)	0.58 (0.52, 0.64)

^a^ Model 1 Adjusted for age (< 60, 60-69.9, 70-79.9, ≥ 80 years old), sex (men, women), education levels (not graduate from high school, graduated from high school, attended college or technical school, graduated from college or technical school, missing), race (non-Hispanic White, non-Hispanic Black, Hispanic, other, missing). ^b^ Model 2: Additionally adjusted for marital status (married or living as married, other, missing), annual household income (<$15,000, $15,000–49,999, ≥$50,000, missing), BMI (kg/m^2^), current smoker (yes, no, missing), current drinker (yes, no, missing), diabetes (yes, no, missing), hypertension (yes, no, missing). * Weighted percentage. *P* < 0.0001 for all

Physical energy expenditure significantly explained 39.0% of the association between gardening and SCD and 58.0% of the association between gardening and SCD-related functional limitations (Table 3). Similar results were obtained when using PA levels (31.3% for SCD; 52.1% for SCD-related functional limitations) as a mediator. Noteworthily, the associations between gardening and SCD (21.5%), and between gardening and SCD-related functional limitations (14.9%) could be attributed to participants’ depressive status. Interestingly, only a small but significant proportion of the gardening-SCD association was explained by higher consumption of fruits and vegetables (3.4%; Table 3). Due to the association of gardening with lower odds of diabetes [20], we further estimated the mediation proportion of diabetes, which was 4.3% for SCD and 2.5% for SCD-related functional limitations.

**Table 3:** Mediation analyses of gardening with subjective cognitive decline (SCD) and SCD-related functional limitations (gardeners vs. non-exercisers, *N* = 54,551), Behavioral Risk Factor Surveillance System 2019. ^a^

Outcomes	Mediators	Gardeners vs. non-exercisers		
		Direct effect (Not adjusted for mediators)	Indirect effect (Adjusted for mediators)	Mediation proportion, %
**SCD**	Energy expenditure ^b^	0.72 (0.68, 0.77)	0.82 (0.70, 0.96)	39.0 (8.2, 82.0)
	Depression ^c^	0.72 (0.68, 0.77)	0.77 (0.73, 0.82)	21.5 (16.7, 27.3)
	Consumptions of fruits and vegetables ^d^	0.72 (0.68, 0.77)	0.73 (0.69, 0.78)	3.4 (1.9, 6.1)
**SCD-related functional limitations**	Energy expenditure	0.49 (0.44, 0.54)	0.74 (0.57, 0.96)	58.0 (25.7, 84.6)
	Depression	0.49 (0.44, 0.54)	0.54 (0.49, 0.60)	14.9 (11.9, 18.6)
	Consumptions of fruits and vegetables	0.49 (0.44, 0.54)	0.49 (0.44, 0.55)	1.6 (0.7, 3.4)

^a^ A multivariate model was adjusted for age (< 60, 60-69.9, 70-79.9, ≥ 80 years old), sex (men, women), education levels (not graduate from high school, graduated from high school, attended college or technical school, graduated from college or technical school, missing), race (non-Hispanic White, non-Hispanic Black, Hispanic, other), marital status (married or living as married, other), annual household income (<$15,000, $15,000–49,999, ≥$50,000, missing), BMI (kg/m^2^), current smoker (yes, no, missing), current drinker (yes, no, missing), diabetes (yes, no, missing), hypertension (yes, no, missing). ^b^ Energy expenditure: product of the MET value of each activity and the corresponding weekly duration (MET-hours/week). ^c^ Depression: perceived depression or not. ^d^ Consumptions of fruits and vegetables: total frequency of participants’ daily intake of fruits, dark green vegetables, potatoes, and other vegetables. *P* < 0.01 for all

The interactions between gardening and each stratifying variable (age, sex, education levels, race, and BMI) were not significant (Table 4). Similar inverse associations of gardening with SCD and SCD-related functional limitations persisted in each subgroup. Sensitivity analysis, after excluding the gardeners who had higher PA levels from other exercises or stratifying by the 32 states, generated similar results (Supplemental Tables 2, 3).

**Table 4:** Association of gardening with subjective cognitive decline (SCD) and SCD-related functional limitations stratified by SCD-related risk factors (gardeners vs. non-exercisers, *N* = 54,551), Behavioral Risk Factor Surveillance System 2019. ^a^

Variables	Participants	Subjective cognitive decline (SCD)			SCD-related functional limitations		
		Non-exercisers	Gardeners	*P*-interaction	Non-exercisers	Gardeners	*P*-interaction
**Age**				0.36			0.42
< 60	15,814	1 (Ref)	0.69 (0.54, 0.89) *		1 (Ref)	0.60 (0.41, 0.88) *	
≥ 60	38,019	1 (Ref)	0.72 (0.61, 0.85) *		1 (Ref)	0.49 (0.35, 0.69) *	
**Sex**				0.61			0.68
Men	21,061	1 (Ref)	0.73 (0.59, 0.91) *		1 (Ref)	0.50 (0.35, 0.73) *	
Women	33,490	1 (Ref)	0.70 (0.58, 0.84) *		1 (Ref)	0.55 (0.39, 0.76) *	
**Education levels**				0.94			0.22
Below college	24,244	1 (Ref)	0.71 (0.56, 0.90) *		1 (Ref)	0.60 (0.41, 0.88) *	
Attended college	30,115	1 (Ref)	0.71 (0.59, 0.85) *		1 (Ref)	0.46 (0.34, 0.62) *	
**Race**				0.66			0.21
Non-Hispanic White	43,670	1 (Ref)	0.70 (0.61, 0.81) *		1 (Ref)	0.49 (0.37, 0.64) *	
Other	9892	1 (Ref)	0.75 (0.49, 1.15)		1 (Ref)	0.66 (0.38, 1.16) *	
**Body mass index**				0.45			0.58
<25.0	13,147	1 (Ref)	0.67 (0.52, 0.85) *		1 (Ref)	0.46 (0.32, 0.67) *	
≥ 25.0	41,404	1 (Ref)	0.72 (0.60, 0.86) *		1 (Ref)	0.54 (0.39, 0.75) *	

^a^ A multivariate model was adjusted for age (< 60, 60-69.9, 70-79.9, ≥ 80 years old, missing), sex (men, women), education levels (not graduate from high school, graduated from high school, attended college or technical school, graduated from college or technical school, missing), race (non-Hispanic White, non-Hispanic Black, Hispanic, other, missing), marital status (married or living as married, other, missing), annual household income (<$15,000, $15,000–49,999, ≥$50,000, missing), BMI (kg/m^2^), current smoker (yes, no, missing), current drinker (yes, no, missing), diabetes (yes, no, missing), hypertension (yes, no, missing). *Compared with the non-exercisers group: *P* < 0.01. Missing data were not presented

## Discussion

In this large, nationally representative sample of US adults aged 45 + years, gardening was significantly associated with lower odds of having SCD and SCD-related functional limitations. Similar patterns were observed among participants when stratified by age, sex, socioeconomic levels, and obesity status. The effect size of gardening on SCD was comparable with that of other exercisers on SCD. Mechanistically, energy expenditure and depressive status was identified as the critical mediators for the association of gardening with SCD and SCD-related functional limitations.

The significant association between gardening and SCD was consistent with a previous meta-analysis of PA and dementia [37], with the additional analyses exploring pathways for the potential impact of gardening on cognition. This meta-analysis pooled previous cohort studies [22–24] and reported similar inverse associations between gardening (OR 0.59, 95% CI 0.50–0.70), and regular exercise (OR 0.58, 95% CI 0.47–0.72) with all-cause dementia risk [37]. A recent cohort study of 4,564 older Japanese adults also reported that field work or gardening was associated with ~ 30% lower risk of dementia compared with non-exercisers, for women and the individuals aged 65–74 years in particular [25], however, insufficient representativeness might result from non-random recruitment of the participants might exist in this study. Consistently, a systematic meta-analysis including four RCTs and ten quasi-experimental studies identified a significant increase in cognitive function scores in the populations with dementia who engaged in participatory horticultural therapy [38], which provided empirical evidence supporting the effectiveness of gardening in ameliorating cognitive impairment. Despite insufficient adjustment of covariates in the models and limitations of small sample sizes might be of concern, their results still supported our findings regarding the inverse association of gardening with SCD and SCD-related functional limitations. Additionally, the previous studies investigating the gardening-dementia association commonly concentrated on older adults aged 60 + years [22–25]. The present study included the much younger populations, aged 45–60 years, for the SCD investigation. Therefore, it is possible that participation in gardening could result in the early suppression and prevention of SCD and even latent dementia in middle-aged and older populations.

We observed that gardeners had a 28% lower odd of SCD and a 43% lower odd of SCD-related functional limitations using a nationally larger-scale community-based population after controlling for the potential confounders. Interestingly, a large proportion (~ 15–22%) of the associations between gardening and SCD or SCD-related functional limitations was explained by participants’ depressive status. Depressive disorder was a well-established risk factor for cognitive decline and was commonly observed in individuals with cognitive impairment [39–41]. For example, a recent cross-sectional study of 191,054 individuals from Korea reported higher risks of SCD (OR 3.11, 95% CI 2.87–3.36) and SCD-related functional limitations (OR 5.47, 95% CI 4.85–6.17) associated with depressive symptoms [42]. In addition to cognitive benefits, gardening was suggested to promote psychological and spiritual well-being. For instance, involvement in community gardening may offer an enjoyable experience to the participants and improve neuroendocrine production, thereby promote subjective happiness and affective restoration from stress and depressive symptoms [43–46]. Intriguingly, mental health status varied by the length of gardening experience and time spent on gardening [47]. The more stressed and anxious the individuals were, the greater their mental health status gardening improved [46].

As expected, we found that physical energy expenditure mediated approximately half of the gardening-cognition association. In line with our results, a study using the 2015 BRFSS data also reported an inversely dose-response relationship between activity levels and SCD [32]. When further studying SCD-related functional limitations, we observed a stronger effect size of mediation, relative to SCD. This could be explained by the additional benefits of gardening on physical functioning. However, we cannot exclude the possibility of reverse causality -- individuals with fewer functional limitations had more opportunities to engage in PA and therefore had higher energy expenditure.

Limitations of the study existed. First, our study had the general cross-sectional design limitations, such as restricted causal inference due to the lack of temporal association between exposure and outcomes. Second, all information was self-reported and recall bias could exist, which may be susceptible to misclassification of the variables, in particular SCD, SCD-related functional limitations, depressive status, frequency and duration of each PA. However, the verified reliability and validity of BRFSS data [48] might partially address this concern, with incorporation of cell phone data, credible weighting methods, and well-trained and experienced BRFSS interviewers. Third, we speculated that selection bias might exist in our study, because the proportions of individuals aged ≥ 60 years (62.8%), men (50.5%), and highly educated participants (87.8%), other exercisers (71.8%) in the excluded participants with missing cognitive decline information (*n* = 72,100) were higher than those included (*n* = 136,748) (Supplemental Table 4). Fourth, despite efforts to control for covariates, unaccounted-for confounding factors still remain and residual confounding is thus of concern. We failed to adjust for some important confounders, such as other commonly encountered diseases (e.g. stroke) [49], and family history of dementia [50]. Finally, we did not explore the association of gardening types with SCD and SCD-related limitations, because the gardening types cannot be subdivided in the BRFSS.

## Conclusions

 Based on a large nationally representative population of US adults, we observed that gardening was significantly associated with better cognitive performance, as were other exercises. The observed inverse association was predominantly attributed to higher energy expenditure and alleviated depressive status. Further studies are warranted to replicate our findings and explore the underlying mechanisms in depth.

### Electronic supplementary material

Below is the link to the electronic supplementary material.

Supplementary Material 1

## Data Availability

All data requests should be submitted to the corresponding author (Prof. Xiang Gao; xiang_gao@fudan.edu.cn) for consideration.
