# Supplementary material for: Gardening and subjective cognitive decline: a cross-sectional study and mediation analyses of 136,748 adults aged 45+ years
Source: Nutr J. 2024 Jun 4;23:59. doi: 10.1186/s12937-024-00959-9 (PMC11149237; doi:10.1186/s12937-024-00959-9)
Supplement: Supplementary file 1 — Supplementary Material 1 [file 12937_2024_959_MOESM1_ESM.docx]

**Supplementary Tables**

**Supplementary Table 1.** Prevalence ratios and 95% confidence interval of subjective cognitive decline (SCD) and SCD-related functional limitations according to physical activity types.

**Supplementary Table 2.** Sensitivity analysis [odds ratio (OR) and 95% confidence interval (CI)] after excluding the gardeners who engaged in other activities for ≥150 minutes.

**Supplemental Table 3.** Sensitivity analysis [odds ratio (OR) and 95% confidence interval (CI)] after stratifying the population by states.

**Supplementary Table 4.** Weighted proportion of age, sex, and physical activity types comparing the excluded participants with missing cognitive decline information (n=72,100) to those included (n=136,748).

**Supplementary Table** **1. Prevalence ratios** **and 95% confidence interval of subjective cognitive decline (SCD) and SCD-related functional limitations according to physical activity types.**

|  | **Non-exercisers** | **Gardeners** | **Other exercisers** |
| --- | --- | --- | --- |
| **SCD** |  |  |  |
| Model 1 ^a^ | 1 (Ref) | 0.65 (0.61, 0.69) * | 0.66 (0.64, 0.69) * |
| Model 2 ^b^ | 1 (Ref) | 0.74 (0.70, 0.79) * | 0.76 (0.73, 0.78) * |
| **SCD-related functional limitations** |  |  |  |
| Model 1 | 1 (Ref) | 0.41 (0.37, 0.46) * | 0.49 (0.46, 0.51) * |
| Model 2 | 1 (Ref) | 0.65 (0.59, 0.71) * | 0.67 (0.64, 0.70) * |

^a^ Model 1: Adjusted for age (<60, 60-69.9, 70-79.9, ≥80 years old), sex (men, women), education levels (not graduate from high school, graduated from high school, attended college or technical school, graduated from college or technical school, missing), race (non-Hispanic White, non-Hispanic Black, Hispanic, other, missing). ^b^ Model 2: Additionally adjusted for marital status (married or living as married, other, missing), annual household income (＜$15000, $15000-49999, ≥$50000, missing), BMI (kg/m^2^), current smoker (yes, no, missing), current drinker (yes, no, missing), diabetes (yes, no, missing), hypertension (yes, no, missing). *Compared with the non-exercisers group: *P*<0.0001.

**Supplementary Table 2. Sensitivity analysis [odds ratio (OR) and 95% confidence interval (CI)] after excluding the gardeners who engaged in other activities for ≥150 minutes.**

|  | **Non-exercisers** | **Gardeners** |
| --- | --- | --- |
| **Individuals with SCD / total, % *** | 6357/41846 (15.9) | 566/6016 (9.9) |
| OR (95%CI) ^a^ | 1 (Ref) | 0.77 (0.63, 0.93) |
| **Individuals with SCD-related functional limitations / total, % *** | 3512/41846 (9.3) | 196/6016 (4.3) |
| OR (95%CI) ^a^ | 1 (Ref) | 0.66 (0.46, 0.94) |

^a^ A multivariate model was adjusted for age (<60, 60-69.9, 70-79.9, ≥80 years old, missing), sex (men, women), education levels (not graduate from high school, graduated from high school, attended college or technical school, graduated from college or technical school, missing), race (non-Hispanic White, non-Hispanic Black, Hispanic, other, missing), marital status (married or living as married, other, missing), annual household income (＜$15000, $15000-49999, ≥$50000, missing), BMI (kg/m^2^), current smoker (yes, no, missing), current drinker (yes, no, missing), diabetes (yes, no, missing), hypertension (yes, no, missing). * Weighted percentage. *P*<0.05 for all.

**Supplemental Table 3.** **Sensitivity analysis [odds ratio (OR) and 95% confidence interval (CI)] after stratifying the population by states (gardeners vs. non-exercisers). ^a^**

|  |  | **Subjective cognitive decline (SCD)** | | **SCD-related functional limitations** | |
| --- | --- | --- | --- | --- | --- |
| **States** | **Participants** | **Non-exercisers** | **Gardeners** | **Non-exercisers** | **Gardeners** |
| Alabama | 2026 | 1 (Ref) | 0.48 (0.30, 0.77) | 1 (Ref) | 0.35 (0.18, 0.70) |
| Connecticut | 1968 | 1 (Ref) | 0.64 (0.41, 1.01) | 1 (Ref) | 0.39 (0.17, 0.94) |
| District of Columbia | 372 | 1 (Ref) | 0.41 (0.10, 1.75) | 1 (Ref) | 0.29 (0.02, 4.26) |
| Florida | 4402 | 1 (Ref) | 0.97 (0.62, 1.54) | 1 (Ref) | 0.52 (0.20, 1.35) |
| Georgia | 1724 | 1 (Ref) | 0.48 (0.29, 0.78) | 1 (Ref) | 0.21 (0.09, 0.46) |
| Indiana | 2354 | 1 (Ref) | 0.94 (0.64, 1.39) | 1 (Ref) | 0.65 (0.35, 1.19) |
| Iowa | 2176 | 1 (Ref) | 0.74 (0.48, 1.13) | 1 (Ref) | 0.56 (0.30, 1.05) |
| Kansas | 1336 | 1 (Ref) | 0.63 (0.35, 1.13) | 1 (Ref) | 0.37 (0.11, 1.23) |
| Louisiana | 1191 | 1 (Ref) | 0.86 (0.52, 1.43) | 1 (Ref) | 0.85 (0.43, 1.71) |
| Maryland | 1390 | 1 (Ref) | 0.56 (0.32, 0.99) | 1 (Ref) | 0.38 (0.13, 1.06) |
| Michigan | 749 | 1 (Ref) | 0.53 (0.23, 1.18) | 1 (Ref) | 0.53 (0.15, 1.86) |
| Minnesota | 2827 | 1 (Ref) | 0.59 (0.40, 0.88) | 1 (Ref) | 0.42 (0.22, 0.81) |
| Mississippi | 1740 | 1 (Ref) | 0.34 (0.16, 0.69) | 1 (Ref) | 0.21 (0.07, 0.63) |
| Missouri | 2047 | 1 (Ref) | 0.37 (0.19, 0.71) | 1 (Ref) | 0.39 (0.15, 0.99) |
| Nebraska | 1966 | 1 (Ref) | 1.34 (0.78, 2.31) | 1 (Ref) | 0.92 (0.37, 2.30) |
| Nevada | 504 | 1 (Ref) | 0.52 (0.19, 1.37) | 1 (Ref) | 0.37 (0.07, 1.91) |
| New Mexico | 1400 | 1 (Ref) | 0.63 (0.37, 1.07) | 1 (Ref) | 0.47 (0.19, 1.13) |
| New York | 1033 | 1 (Ref) | 0.55 (0.24, 1.27) | 1 (Ref) | 0.70 (0.20, 2.38) |
| North Dakota | 1527 | 1 (Ref) | 0.34 (0.18, 0.65) | 1 (Ref) | 0.32 (0.08, 1.23) |
| Ohio | 2694 | 1 (Ref) | 0.72 (0.46, 1.12) | 1 (Ref) | 0.35 (0.16, 0.77) |
| Oklahoma | 859 | 1 (Ref) | 0.37 (0.15, 0.87) | 1 (Ref) | 0.37 (0.10, 1.46) |
| Oregon | 1297 | 1 (Ref) | 0.40 (0.24, 0.65) | 1 (Ref) | 0.55 (0.26, 1.17) |
| Pennsylvania | 1356 | 1 (Ref) | 0.81 (0.42, 1.56) | 1 (Ref) | 0.48 (0.14, 1.74) |
| Rhode Island | 1390 | 1 (Ref) | 1.01 (0.59, 1.75) | 1 (Ref) | 0.87 (0.36, 2.06) |
| South Carolina | 1881 | 1 (Ref) | 0.74 (0.41, 1.33) | 1 (Ref) | 0.68 (0.24, 1.92) |
| South Dakota | 1809 | 1 (Ref) | 0.57 (0.20, 1.62) | 1 (Ref) | 0.07 (0.02, 0.25) |
| Tennessee | 1638 | 1 (Ref) | 0.71 (0.44, 1.14) | 1 (Ref) | 0.36 (0.17, 0.79) |
| Texas | 2959 | 1 (Ref) | 0.76 (0.44, 1.31) | 1 (Ref) | 0.70 (0.30, 1.60) |
| Utah | 985 | 1 (Ref) | 0.81 (0.47, 1.38) | 1 (Ref) | 0.26 (0.09, 0.74) |
| Virginia | 2253 | 1 (Ref) | 0.64 (0.41, 0.99) | 1 (Ref) | 0.53 (0.27, 1.05) |
| West Virginia | 1571 | 1 (Ref) | 0.29 (0.15, 0.58) | 1 (Ref) | 0.17 (0.06, 0.53) |
| Wisconsin | 1127 | 1 (Ref) | 0.68 (0.35, 1.35) | 1 (Ref) | 0.21 (0.07, 0.64) |

^a^ A multivariate model was adjusted for age (<60, 60-69.9, 70-79.9, ≥80 years old, missing), sex (men, women), education levels (not graduate from high school, graduated from high school, attended college or technical school, graduated from college or technical school, missing), race (non-Hispanic White, non-Hispanic Black, Hispanic, other, missing), marital status (married or living as married, other, missing), annual household income (＜$15000, $15000-49999, ≥$50000, missing), BMI (kg/m^2^), current smoker (yes, no, missing), current drinker (yes, no, missing), diabetes (yes, no, missing), hypertension (yes, no, missing). *Compared with the non-exercisers group: *P*<0.01. Missing data were not presented.

**Supplementary Table 4. Comparison of age, sex, and physical activity types between the excluded participants with missing cognitive decline information (n=72,100) and those included (n=136,748).**

| **Variables** | **Participants** | **Weighted proportion** | | **Odds ratio (95% confidence interval) *** | |
| --- | --- | --- | --- | --- | --- |
|  |  | **Excluded, %** | **Included, %** | **Excluded** | **Included** |
| **Age, years, %** |  |  |  |  |  |
| <60 | 72555 | 36.3 | 41.5 | 1 (Ref) | 1.25 (1.21, 1.30) |
| ≥60 | 133074 | 62.8 | 56.4 | 1 (Ref) | 0.77 (0.74, 0.79) |
| **Sex, %** |  |  |  |  |  |
| Men | 93712 | 50.5 | 46.4 | 1 (Ref) | 0.84 (0.81, 0.87) |
| Women | 114688 | 49.5 | 53.6 | 1 (Ref) | 1.20 (1.15, 1.24) |
| **Education, %** |  |  |  |  |  |
| Below college | 15020 | 11.7 | 12.5 | 1 (Ref) | 1.12 (1.05, 1.20) |
| Attended college | 192706 | 87.8 | 87.1 | 1 (Ref) | 0.91 (0.85, 0.98) |
| **Physical activity types, %** |  |  |  |  |  |
| Non-exercisers | 59342 | 24.6 | 31.2 | 1 (Ref) | 1.41 (1.36, 1.47) |
| Gardeners | 15792 | 3.6 | 8.8 | 1 (Ref) | 2.50 (2.30, 2.71) |
| Other exercisers | 133266 | 71.8 | 60.0 | 1 (Ref) | 0.58 (0.56, 0.61) |

Weighted percentage. * *P*<0.05 for all.
